# Oral Administration of *Crocus sativus* Tepals Extract Restores High‐Fat Diet‐Induced Gut Dysbiosis and Modulates Intestinal Inflammation and Hepatic Lipid Metabolism

**DOI:** 10.1002/biof.70083

**Published:** 2026-02-11

**Authors:** Biljana Bursać, Miloš Vratarić, Ljupka Gligorovska, Luisa Bellachioma, Ana Teofilović, Danijela Vojnović Milutinović, Camilla Morresi, Elisabetta Damiani, Tiziana Bacchetti, Ana Djordjevic

**Affiliations:** ^1^ Department of Biochemistry Institute for Biological Research “Siniša Stanković”‐National Institute of the Republic of Serbia, University of Belgrade Belgrade Serbia; ^2^ Department of Life and Environmental Sciences Marche Polytechnic University Ancona Italy

**Keywords:** *Crocus sativus*, gut microbiota, inflammation, jejunum, lipid metabolism, liver

## Abstract

Metabolic diseases have increased worldwide in recent decades, mainly due to a sedentary lifestyle and an unhealthy diet, with diet identified as an important regulator of gut microbiota composition. The use of natural products, such as *Crocus sativus* tepals extract (CTE) could be a promising approach to alleviate metabolic disorders. The aim was to investigate the potential ameliorative mechanisms of CTE in metabolic disorders induced by a high‐fat diet in an animal model, focusing on the composition of the gut microbiota and its relationship with the gut‐liver axis. We analyzed liver‐related biochemical and morphological parameters in mice fed a 60% fat diet for 14 weeks and orally treated with CTE during the last 5 weeks of the diet. In addition, jejunal and liver histology, intestinal barrier integrity, inflammation and oxidative stress, liver inflammation and lipid metabolism were investigated. The results showed that oral administration of CTE restored the composition of the gut microbiota and specifically promoted short‐chain fatty acids‐producing and anti‐inflammatory bacterial genera. It also improved intestinal barrier integrity and reduced inflammation in the jejunum and liver, along with a suppression of *Fas* and *CerS6* expression in the liver and a reduction in circulating free fatty acids and β‐hydroxybutyrate levels. Our results indicate a possible link between the gut microbiota and the metabolic benefits of treatment with CTE, suggesting its therapeutic potential for the prevention or treatment of metabolic disorders.

## Graphical abstract

This study shows that oral administration of *Crocus sativus* tepals extract (CTE) restores gut microbiota composition and promotes SCFA‐producing and anti‐inflammatory bacterial genera. It improves intestinal barrier integrity and reduces inflammation in the jejunum and liver, suggesting a possible link between the gut microbiota and the metabolic benefits of CTE treatment.

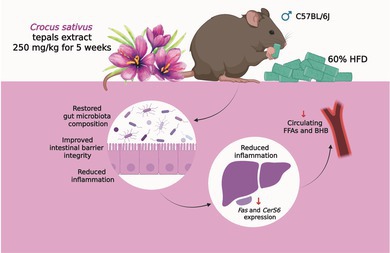

**Abbreviations**
ACC: acetyl‐CoA carboxylase
ALT: alanine aminotransferase
AST: aspartate aminotransferase
BHB: beta‐hydroxybutyrate
Cd36: cluster of differentiation 36
CerS6: ceramide synthase 6
CTE:
*Crocus sativus* tepals extract
DGAT: diacylglycerol O‐acyltransferase
FAS: fatty acid synthase
FFA: free fatty acids
GGT: gamma‐glutamyl transferase
GPX: glutathione peroxidase
GSR: glutathione reductase
HFD: high‐fat diet
Hprt: hypoxanthine‐guanine phosphoribosyl transferase
IL‐1β: interleukin‐1β
IL‐6: interleukin‐6
LPS: lipopolysaccharides
MASLD: metabolic dysfunction‐associated steatotic liver disease
MyD88: myeloid differentiation primary response 88
PGC‐1α: peroxisome proliferator‐activated receptor gamma coactivator 1‐alpha
PPARα: peroxisome proliferator‐activated receptor α
ROS: reactive oxygen species
Scd1: stearoyl‐CoA desaturase‐1
SCFA: short‐chain fatty acid
SOD: superoxide dismutase
SREBP‐1c: sterol regulatory element‐binding protein 1c
TLRs: Toll‐like receptors
TNF‐α: tumor necrosis factor‐α
ZO‐1: zonula occludens 1

## 1 Introduction

Metabolic diseases, including obesity, metabolic dysfunction‐associated steatotic liver disease (MASLD), dyslipidemia, and type 2 diabetes, have shown a rising global prevalence over the past decades, driven largely by sedentary lifestyles, unhealthy diets, and urbanization trends [1]. These diseases share underlying disturbances in insulin sensitivity, lipid metabolism, inflammation, and gut microbiota composition [2], and significantly affect individuals across all age groups, placing a substantial burden on healthcare systems worldwide [3].

Among the modifiable factors, diet has been identified as a key regulator of gut microbiota composition. Excessive dietary fat intake can alter the gut microbial community, leading to gut dysbiosis characterized by reduced bacterial diversity and richness [4]. The state of dysbiosis promotes hepatic lipid accumulation by increasing energy harvest, altering microbial metabolites, and disrupting bile acid signaling, thereby enhancing *de novo* lipogenesis in hepatocytes [5]. Dysbiosis has also been shown to disrupt the integrity of the intestinal barrier by downregulating the expression of tight junction proteins, such as zonula occludens 1 (ZO‐1) and occludin, resulting in increased gut permeability [6]. Increased gut permeability allows translocation of bacterial products, such as lipopolysaccharides (LPS) to the liver, where they activate Toll‐like receptor (TLR) signaling via the myeloid differentiation primary response 88 (MyD88) pathway, inducing the production and release of pro‐inflammatory cytokines, including tumor necrosis factor‐α (TNF‐α), interleukin‐6 (IL‐6), and interleukin‐1β (IL‐1β). Sustained inflammation promotes hepatic lipid accumulation and oxidative stress, contributing to liver damage characteristic of hepatic steatosis [7, 8, 9]. On the other hand, both inflammation and oxidative stress can also be a consequence of lipid accumulation in the liver. Lipid accumulation begins with free fatty acid (FFA) uptake or *de novo* lipogenesis, regulated by sterol regulatory element‐binding protein 1c (SREBP‐1c) and downstream enzymes, acetyl‐CoA carboxylase (ACC) and fatty acid synthase (FAS). Fatty acids are then stored as triglycerides via diacylglycerol O‐acyltransferase (DGAT) or undergo mitochondrial β‐oxidation, controlled by peroxisome proliferator‐activated receptor alpha (PPARα) [10, 11]. Which process prevails depends on the hepatic influx of FFAs. If the influx exceeds the liver's capacity for safe storage or export, hepatocytes accumulate lipotoxic lipid species such as diacylglycerols, ceramides, and long‐chain acylcarnitines [12]. To accommodate lipid overload, hepatocytes upregulate mitochondrial β‐oxidation to prevent lipid accumulation, increasing electron flux through the electron transport chain and promoting excessive production of reactive oxygen species (ROS) [13]. ROS act as potent signaling molecules that activate multiple pro‐inflammatory pathways, especially the NLRP3 inflammasome and redox‐sensitive transcription factors such as nuclear factor‐κB (NF‐κB), further driving transcription of pro‐inflammatory cytokines [14].

Current treatment approaches primarily involve lifestyle modifications and pharmacological interventions. Although available drugs, such as metformin, GLP‐1 receptor agonists, SGLT2 inhibitors, statins, and fibrates provide proven benefits for glycemic and lipid control and weight loss, their use is somewhat limited by high costs and adverse effects such as gastrointestinal complications, vitamin B12 deficiency, anemia, myopathy, and venous thrombosis [15, 16]. Furthermore, these drugs are designed to primarily target single metabolic pathways and may not fully address the gut‐liver‐adipose tissue axis [17]. Thus, there is growing interest worldwide in the use of herbal medicines, mainly due to their natural origin and low side effects [18]. While more studies are needed to compare plant‐derived products with clinical drugs to determine how bioactive plant resources may complement or enhance existing metabolic disease therapies, experimental evidence suggests that plant extracts can beneficially modulate gut microbiota, oxidative stress, and inflammatory pathways, offering multi‐target actions with generally favorable safety profiles [19]. For example, a recent study showed that *Ligustrum robustum* extract improves glucose and lipid homeostasis and reshapes gut microbial structure in mice fed a Western diet [20]. Similarly, *Dracocephalum moldavica* tea has been shown to alleviate high‐fat diet‐induced hyperlipidemia in rats through regulation of microbiota and lipid metabolism [21], while hesperetin, a citrus flavonoid, may be an effective dietary supplement for improving MASLD by suppressing hepatic oxidative stress and inflammation [22]. Both myricetin, a flavonoid found in onions, berries, grapes, and red wine, and oroxin B, a constituent of the *Oroxylum indicum* plant, reduce hepatic inflammation and oxidative damage primarily by modulating gut microbiota [23, 24]. A recent review highlights multiple plants with potential protective effects in diabetic nephropathy, achieved through anti‐inflammatory and microbiota‐mediated mechanisms [25]. Saffron, a spice obtained from the stigmas of the *Crocus sativus* plant, a member of the *Iridaceae* family, has also shown promise as an anti‐obesity agent. It has positive effects on insulin sensitivity as well as anti‐inflammatory, antioxidant, lipid‐ and glucose‐lowering properties [26, 27, 28, 29]. Recently, increasing attention has been directed towards the role of saffron in metabolic regulation, as crocin‐1, the main bioactive compound in saffron, has been shown to influence the composition of the gut microbiota [30].

Although previous studies have shown the protective effect of *Crocus sativus* stigmas extract against metabolic disorders, including antioxidant and anti‐inflammatory effects [28, 31, 32], the biological activity of *Crocus sativus* tepals extracts (CTE), a traditionally discarded by‐product of saffron production, is still largely unexplored. *Crocus sativus* tepals constitute the majority of plant material discarded during saffron spice production, as approximately 53 kg of tepals are wasted for every 1 kg of dried stigmas. Tepals are a rich source of bioactives, mainly flavonoids (flavones, flavonols, and flavanones), with kaempferol glycosides and anthocyanins being the most abundant [33]. These compounds exhibit antioxidant, hepatoprotective, cardioprotective, antidiabetic, antimicrobial, and wound‐healing activities [34, 35, 36, 37, 38, 39]. Drug‐like and pharmacokinetic properties of compounds from North African *Crocus sativus* extracts were assessed using PASS, showing a favorable safety profile with no mutagenicity and high LD_50_ values [40]. The Ames test also confirmed non‐mutagenicity of CTE [38], supporting its potential use in pharmaceutical, nutraceutical, and cosmetic applications, though further in vivo validation is needed.

Recently, we reported the ability of CTE to suppress adipose tissue hypertrophy and to improve systemic insulin resistance in mice fed a high‐fat diet [41], however, the effects of extract from tepals in the context of regulating the microbiota‐gut‐liver axis remain unknown. Given the central role of the gut microbiota in regulating intestinal barrier integrity, immune responses, and metabolic pathways [42] investigating the complex interactions within the microbiota‐gut‐liver axis represents a promising approach to better understand the development and progression of obesity‐related metabolic disorders. Therefore, the aim of the present study was to elucidate the potential ameliorative mechanisms of CTE on metabolic disturbances evoked by the high‐fat diet in an animal model, focusing primarily on gut microbiota composition and its relationship to the gut‐liver axis. To this end, we analyzed biochemical and morphological parameters in mice fed a 60% fat diet for 14 weeks and orally treated with CTE during the final 5 weeks of the diet. In addition, gut histology, barrier integrity, gut inflammation, and oxidative stress, as well as liver inflammation and lipid metabolism, were examined.

## 2 Experimental Procedures

### 2.1 CTE Preparation and Characterization

The preparation and characterization of CTE was performed as previously described [41]. Briefly, flowers were provided by a local farm “Tesoro delle Api” (Sant'Elpidio a Mare, FM, Italy) and were cultivated without any chemical treatment. The tepals were manually separated and frozen at −20°C before being lyophilized in a freeze dryer (LYOQUEST‐55, Seneco, Italy). The tepals extract was prepared using a controlled ethanol/water (80/20 v/v) extraction method, as previously described [41]. The extract was then purified and concentrated and subsequently stored at −20°C until use. Metabolomics profiling and compounds identified in CTEs through U‐HPLC‐HRMS technique were previously described in detail [41].

### 2.2 Animals, Treatment and Experimental Design

At the beginning of the experiment, male C57BL/6J mice (2.5 months old) were randomly divided into three experimental groups: control (C) group (*n* = 11), high‐fat diet (HFD) group (*n* = 10) and high‐fat diet with CTE (HFD + CTE) group (*n* = 9). During the experiment, two animals were housed in a cage separated by a perforated, transparent acrylic partition so that they were not socially isolated and could communicate with each other. The animals were housed under standard conditions at 22°C± 2°C with a 12‐h light/dark cycle, constant humidity and free access to water, with constant veterinary care. Group C had *ad libitum* access to a control diet (rodent diet with 10 kcal% fat, D12450J, Research diets, New Brunswick, USA), while the HFD and HFD + CTE groups had *ad libitum* access to rodent diet with 60 kcal% fat (D12492, Research diets, New Brunswick, USA) for 14 weeks. During the last 5 weeks of the high‐fat diet, the HFD + CTE group started receiving CTE dissolved in phosphate‐buffered saline (PBS), while C and HFD groups received only PBS by oral gavage. CTE was administered daily to mice at a dose of 250 mg/kg body mass. The dose was selected based on previous studies in animal models, including our own, which showed that this concentration is both effective and safe in rodents. Specifically, our study demonstrated that oral administration of 250 mg/kg hydroethanolic CTE for 5 weeks led to significant metabolic improvements in a diet‐induced obesity model, including reduced body mass, enhanced systemic insulin sensitivity, decreased triglycerides, and improved lipid peroxidation [41]. Other independent studies have also used this dose and reported beneficial physiological outcomes [43, 44, 45]. This aligns with toxicity data from other studies, which reported maximum non‐fatal doses of tepals aqueous and ethanolic extracts at 3.6 g/kg and 8 g/kg (i.p.), with LD50 values of 6.67 g/kg and 9.99 g/kg, respectively [46]. Based on this body of evidence, 250 mg/kg is a widely used, effective, and safe dose for studying the biological activity of CTE.

With regards to extrapolation to potential human consumption, the human equivalent dose (HED) was calculated using the following formula: HED (mg/kg) = Animal dose (mg/kg) × (Animal Km/Human Km), where Km factor for mouse is 3, while the Km factor for humans is 37 [47]. This calculation results in a HED for CTE of 20 mg/kg, which equates to 1.2 g of tepals extract for a 60 kg person. This value is about 10 times lower compared to the values reported as the safe dose for humans of several “herbal medicines” evaluated by calculating HED from animal‐based toxicity studies [48].

This study was approved by the Ethical Committee for the Use of Laboratory Animals of the Ministry of Agriculture, Forestry and Water Economy of the Republic of Serbia (No. 323‐07‐02390‐2022‐05 from February 28, 2022). All animal experiments were conducted in accordance with EEC Directive 2010/63/EU on the protection of animals used for experimental and other scientific purposes.

### 2.3 FITC‐Dextran Permeability Assay

Intestinal permeability was assessed in vivo by measuring plasma levels of 4 kDa fluorescein isothiocyanate‐dextran (FITC‐dextran, FD4‐1G; Sigma‐Aldrich, St. Louis, USA) 5 days prior to the end of the experiment. Mice were fasted for 4 h before and after oral gavage with 150 μL of FITC‐dextran solution (80 mg/mL). Four hours after administration, blood samples were collected via retro‐orbital bleeding and centrifuged at 3000 × g for 10 min at 4°C. Plasma samples were diluted 1:5 (v/v) in PBS, and fluorescence intensity was measured at 530 nm (excitation at 485 nm) using a Synergy H1 microplate reader (BioTek Instruments, Winooski, VT, USA).

### 2.4 Fecal Sample Processing and Microbial Analysis

Fecal samples from animals were collected in sterile tubes after spontaneous defecation, immediately frozen in liquid nitrogen, and stored until analysis. Genomic DNA from fecal samples was extracted using the Quick‐DNA Fecal/Soil Microbe Miniprep Kit (Zymo Research, Irvine, CA, USA) according to the manufacturer's instructions. The extracted DNA was sent to Novogen Co. (P.R. China) for commercial V3‐V4 16S rRNA paired‐end sequencing. Raw sequencing data were processed using Quantitative Insights into Microbial Ecology 2 (QIIME2, version 2024.5). Barcodes and primers were removed with the q2‐cutadapt plugin, while low‐quality sequences and chimeras were filtered out. Overlapping sequences were merged, and amplicon sequence variants (ASVs) were generated using q2‐dada2. Taxonomic classification was performed with the q2‐feature‐classifier, employing a Naive Bayes model trained on the Silva 138.2 reference database. Data were imported into R (https://www.R‐project.org/) using file2meco and analyzed with the *microeco* package. Alpha diversity metrics, including Observed Features, Shannon Diversity, Pielou Evenness and Fisher Index, were calculated. Beta diversity was assessed using Bray–Curtis dissimilarity and Jaccard, unweighted UniFrac and weighted UniFrac distances, visualized through principal coordinate analysis (PCoA) plots.

### 2.5 Serum and Tissue Preparation and Determination of Biochemical and Morphological Parameters

At the end of the experiment, the animals were killed by rapid decapitation, after 4 h of fasting, and trunk blood was collected for serum preparation. The serum was obtained by low‐speed centrifugation (2000 × g for 10 min) following a 30 min incubation at room temperature. Samples were then stored at −70°C for further analysis. Immediately after decapitation, liver and small intestine (jejunum) samples were isolated from each animal and the livers were weighed.

The concentration of cholesterol, aspartate aminotransferase (AST), alanine aminotransferase (ALT), gamma‐glutamyl transferase (GGT), FFA and beta‐hydroxybutyrate (BHB) were measured on a semi‐automatic biochemistry analyzer Mindray BS‐240 (Mindray, Shenzhen, P.R. China) by using commercially available reagents (cholesterol: 11,505, AST: 11,531, ALT: 11,533, GGT: 11,510, FFA: 11,840, BHB: 12,525, BioSystems, Barcelona, Spain).

### 2.6 Histological and Morphometric Analysis of the Liver and Jejunum

For histological and morphometric assessments, liver and jejunum specimens were fixed in 4% paraformaldehyde for 24 h, dehydrated in an ethanol gradient, cleared in xylene and embedded in paraffin. The paraffin blocks were sectioned into 5 μm thick slices and stained with hematoxylin and eosin using standard protocols. Image acquisition for analysis was performed using a Leitz DMRB light microscope equipped with a Leica MC190 HD camera and Leica Application Suite (LAS) 4.11.0 software (Leica Microsystems, Wetzlar, Germany) at 10× magnification. Morphometric measurements of the jejunum tissue, including villus length, crypt depth, mucosal thickness, submucosal thickness and muscularis externa thickness were conducted using ImageJ software (https://imagej.nih.gov/ij/). The analysis was performed in a blinded manner, with three sections measured per animal (at 100 μm intervals).

### 2.7 RNA Extraction, Reverse Transcription and Real‐Time PCR

Total RNA was extracted from the liver and jejunum using the TRI reagent solution (AM9738, Thermo Fisher Scientific) following the manufacturer's instructions. RNA concentrations were measured using a NanoPhotometer N60 (Implen, Munich, Germany) by assessing optical density at 260 nm. Reverse transcription was carried out using the High‐Capacity cDNA Reverse Transcription Kit (Applied BioSystems, USA) according to the manufacturer's protocol, and the resulting cDNA was stored at −80°C until further use.

To determine mRNA expression levels of target genes, real‐time polymerase chain reaction (PCR) was performed using the Advanced Universal SYBR Green Supermix (Bio‐Rad Laboratories, USA) and Specific primers (Microsynth, Balgach, Switzerland): cluster of differentiation 36 (*Cd36*, F: 5′‐CAT TTG CAG GTC TAT CTA CG‐3′; R: 5′‐CAA TGT CTA GCA CAC CAT AAG‐3′), ceramide synthase 6 (*CerS6*, F: 5′‐GAG ATT AGA AGG GCT CTC CA‐3′; R: 5′‐CAC ATG CTC TCA CAG AAC CT‐3′), *Fas* (F: 5′‐TTG CTG GCA CTA CAG AAT GC‐3′; R: 5′‐AAC AGC CTC AGA GCG ACA AT‐3′), stearoyl‐CoA desaturase‐1 (*Scd1*, F: 5′‐CTG TAC GGG ATC ATA CTG GTT C‐3′; R: 5′‐GCC GTG CCT TGT AAG TTC TG‐3′), *Dgat1* (F: 5′‐GTG CAC AAG TGG TGC ATC AG‐3′; R: 5′‐CAG TGG GAC CTG AGC CAT CA‐3′), toll‐like receptor 4 (*Tlr4*, F: 5′‐ATC ATC CAG GAA GGC TTC CA‐3′; R: 5′‐GCT AAG AAG GCG ATA CAA TTC‐3′), *Myd88* (F: 5′‐TCA TGT TCT CCA TAC CCT TGG T‐3′; R: 5′‐AAA CTG CGA GTG GGG TCA G‐3′), *Tnf‐α* (F: 5′‐CTC AGC CTC TTC TCA TTC CTG CT‐3′; R: 5′‐CTG ATG AGA GGG AGG CCA TT‐3′) and *Il‐1b* (F: 5′‐CAG GCT CCG AGA TGA ACA AC‐3′; R: 5′‐AGG CCA CAG GTA TTT TGT CG‐3′). Normalization of cDNA expression was performed using hypoxanthine‐guanine phosphoribosyl transferase (*Hprt*) as an endogenous control (F: 5′‐TCC TCC TCA GAC CGC TTT T‐3′; R: 5′‐CCT GGT TCA TCA TCG CTA ATC‐3′). All reactions were conducted in duplicate in a total volume of 20 μL, containing 20 ng of cDNA template, using the Quant Studio Real‐Time PCR System (Applied Biosystems, USA). The thermal cycling conditions were as follows: initial incubation at 50°C for 2 min, followed by 10 min at 95°C, then 45 cycles of 95°C for 15 s and 60°C for 60 s. A melting curve analysis was performed to confirm the formation of a single PCR product. Relative gene expression levels were quantified using the comparative 2^−ΔΔCt^ method, where ΔCt represents the difference between the Ct value of the target gene and that of the endogenous control. Data analysis was performed using Quant Studio Design and Analysis software v1.4.0 (Applied Biosystems, USA), with a confidence level of 95% (*p* ≤ 0.05).

### 2.8 Preparation of Protein Fractions From the Jejunum and Liver Tissues

The total protein fraction from the jejunum of each animal was prepared using the TRIzol protocol, following the manufacturer's instructions. After RNA precipitation, ethanol was added to the remaining organic phase, followed by centrifugation at 2000 × g for 5 min at 4°C. The protein fraction was precipitated from the phenol‐ethanol supernatant using acetone and centrifuged at 12,000 × g for 10 min at 4°C. The resulting protein pellets were dissolved in 0.3 M guanidine hydrochloride in 95% ethanol containing 2.5% glycerol, followed by sonication on ice and washing in the same buffer. After protein pelleting by centrifugation at 8000 × g for 5 min at 4°C, pellets were dissolved in lysis buffer (2.5 mM Tris–HCl, pH 6.8, 2% sodium dodecyl sulfate (SDS), 10% glycerol, and 50 mM dithiothreitol (DTT)). Samples were stored at −80°C for further analysis.

For the preparation of cytoplasmic, microsomal and nuclear fractions from the liver, samples from each animal were weighed and homogenized using a Janke‐Kunkel Ultra Turrax homogenizer (30 s homogenization/30 s pause/30 s homogenization) in four volumes (w/v) of ice‐cold homogenization buffer (20 mM Tris–HCl, pH 7.2, 10% glycerol, 50 mM NaCl, 1 mM EDTA‐Na_2_, 1 mM EGTA‐Na_2_, 2 mM DTT and phosphatase and protease inhibitors). The homogenates were filtered through gauze and centrifuged at 2000 × g for 15 min at 4°C (Eppendorf 5804/R, Hamburg, Germany). The obtained supernatants (S1) were further processed to isolate cytoplasmic and microsomal fractions, while the pellets (P1) were used for nuclear fraction isolation. S1 supernatants were centrifuged at 14,000 × g for 30 min at 4°C, and the obtained supernatants (S2) were then ultracentrifuged at 200,000 × g for 90 min at 4°C (Beckman L7‐55, Brea, CA, United States). The resulting pellets (P2) were resuspended and sonicated (3 × 5 s, 1 A, 50/60 Hz) in 50 mM potassium phosphate buffer (pH 7.4) containing 0.1 mM EDTA‐Na2, 20% glycerol and 0.1 mM DTT and used as microsomal fractions, while supernatants were collected as cytoplasmic fractions. For nuclear fraction isolation, the P1 pellets were washed twice in HEPES buffer (25 mM HEPES, pH 7.6, 1 mM EDTA‐Na_2_, 1 mM EGTA‐Na_2_, 10% glycerol, 50 mM NaCl, 2 mM DTT and phosphatase and protease inhibitors) by centrifugation at 4000 × g for 10 min at 4°C. The resulting pellets were resuspended in NUN buffer (25 mM HEPES, pH 7.6, 1 M urea, 300 mM NaCl, 1% Nonidet P‐40 and protease and phosphatase inhibitors), and incubated on ice for 90 min with constant shaking and frequent vortexing. Following centrifugation at 8000 × g for 10 min at 4°C, the resulting supernatants were collected as nuclear fractions. Protein fractions from the jejunum and liver tissues were stored at −80°C for further analysis.

### 2.9 Western Blot Analysis

Protein concentrations from the jejunum and liver fractions were determined using the Lowry method, with bovine serum albumin (BSA) as a standard. The samples were boiled in 2 × Laemmli buffer for 5 min, and 40 μg of protein was subjected to electrophoresis on 7.5% or 12% SDS–polyacrylamide gels. Following electrophoresis, proteins were transferred from the gels onto polyvinylidene difluoride (PVDF) membranes (Immobilon‐FL, Millipore, USA). The membranes were blocked for 1 h with 2% BSA and then incubated overnight at 4°C with specific primary antibodies: anti‐ZO‐1 (1:1000, 40–2200, Thermo Fisher Scientific), anti‐occludin (1:1000, ab216327, Abcam), anti‐catalase (1:1000, FNab01301, FineTest), anti‐glutathione reductase (GSR, 1:2000, FNab03682, FineTest), anti‐glutathione peroxidase 4 (GPX4, 1:2000, FNab10452, FineTest), anti‐superoxide dismutase 1 (SOD1, 1:500, FNab08103, FineTest), anti‐superoxide dismutase 2 (SOD2, 1:5000, FNab08104, FineTest), anti‐SREBP‐1c (1:500, sc‐366, Santa Cruz Biotechnology), anti‐PPARα (1:1000, ab192599, Abcam), anti‐peroxisome proliferator‐activated receptor gamma coactivator 1‐alpha (PGC‐1α, 1:250, sc‐13067, Santa Cruz Biotechnology), anti‐TNF‐α (D2D411948, Cell Signaling 1:1000), anti‐TLR4 (ab22048, Abcam, 1:1000), anti‐IL‐1β (H‐153, Santa Cruz Biotechnology, 1:500), anti‐CERS6 (PA5‐20648, Thermo Fisher Scientific, 1:1000), anti‐DGAT1 (PA5‐117074, Thermo Fisher Scientific, 1:1000), anti‐CD36 (sc‐7309, Santa Cruz Biotechnology, 1:500), and anti‐ACC (Fnab00077, FineTest, 1:1000). Anti‐β‐actin (1:10,000, ab8227, Abcam) was used as a loading control for the total protein extracts and cytosol, anti‐Calnexin (ab22595, Abcam, 1:2000) was used as a loading control for microsomal fractions, while anti‐Lamin B1 (sc‐374015, Santa Cruz Biotechnology, 1:1000) was used for nuclear fractions. Membranes were extensively washed in PBS containing 0.1% Tween‐20 and incubated for 90 min with the corresponding secondary antibodies: mouse (1:30,000, Abcam, ab97046) or rabbit (1:20,000, Abcam, ab6721) horseradish peroxidase (HRP)‐conjugated antibodies. Immunoreactive protein bands were detected using a chemiluminescent method with the iBright CL1500 system (Thermo Fisher Scientific), and quantitative analysis was performed using iBright software.

### 2.10 Statistical Analyses and Data Visualization

Biochemistry, histology, qPCR, and Western blot data are presented as means ± SEM and tested for normality using the Shapiro–Wilk test. Normally distributed data were analyzed using one‐way analysis of variance (ANOVA), followed by Tukey's *post hoc* test. Data that deviated from a normal distribution were analyzed using the non‐parametric Kruskal–Wallis *H* test, followed by Dunn's *post hoc* test. Differences between groups were considered statistically significant at *p* < 0.05. Statistical analyses were performed using GraphPad Prism 8 software (San Diego, USA). ANOSIM analysis was used for beta diversity analysis of microbiota. Data visualization was performed using R programming language (version 4.1.0) and GraphPad Prism 8.

## 3 Results

### 3.1 The Effects on CTE on Liver‐Related Biochemical and Morphological Parameters

As shown in Table 1, CTE supplementation did not affect serum ALT, AST, and GGT levels. Serum FFA level was significantly decreased in the HFD + CTE group compared to the control group (*p* < 0.001), and in line with this, serum BHB level, a marker of lipid oxidation and ketogenesis, was reduced after CTE supplementation compared to the HFD group (*p* < 0.05). Total cholesterol levels were significantly elevated in both HFD (*p* < 0.05) and HFD + CTE group (*p* < 0.01) compared to the control mice (Table 1). Liver mass and the liver‐to‐body mass ratio did not significantly differ between experimental groups.

**TABLE 1:** The effects of high‐fat diet and *Crocus sativus* tepals extract (CTE) on liver‐related biochemical and morphological parameters.

	C (*n* = 11)	HFD (*n* = 10)	HFD + CTE (*n* = 9)
ALT (U/L)	71.08 ± 14.24	89.42 ± 16.25	113.37 ± 24.62
AST (U/L)	371.71 ± 35.05	290.18 ± 24.63	322.59 ± 24.75
GGT (U/L)	2.93 ± 0.42	2.31 ± 0.29	2.52 ± 0.49
FFA (mmol/L)	1.67 ± 0.11	1.38 ± 0.06	1.11 ± 0.08***
BHB (mmol/L)	0.14 ± 0.02	0.19 ± 0.02	0.12 ± 0.01#
Total cholesterol level (mmol/L)	4.00 ± 0.40	5.50 ± 0.32*	5.93 ± 0.18**
Liver mass (g)	1.14 ± 0.05	1.43 ± 0.10	1.39 ± 0.12
Liver mass/body mass ratio (×1000)	33.53 ± 1.03	32.22 ± 2.12	32.29 ± 1.62

*Note:* Alanine transaminase (ALT), aspartate aminotransferase (AST), gamma‐glutamyl transferase (GGT), free fatty acids (FFA), total cholesterol, beta‐hydroxybutyrate (BHB), liver mass and liver to body mass ratio were measured in control group (C), high‐fat diet group (HFD) and high‐fat diet + *Crocus sativus* tepals extract group (HFD + CTE). All data are presented as mean ± SEM. One‐way ANOVA followed by the Tukey *post hoc* test or non‐parametric Kruskal–Wallis *H* test followed by Dunn's *post hoc* test were used to assess statistical significance. Asterisk (*) indicates significant differences in regard to C group (*p < 0.05, **p < 0.01, ***p < 0.001), while hashtag (#) indicates significant differences in regard to HFD group (#*p* < 0.05).

### 3.2 CTE Treatment Restores High‐Fat Diet‐Induced Gut Dysbiosis

**FIGURE 1:**
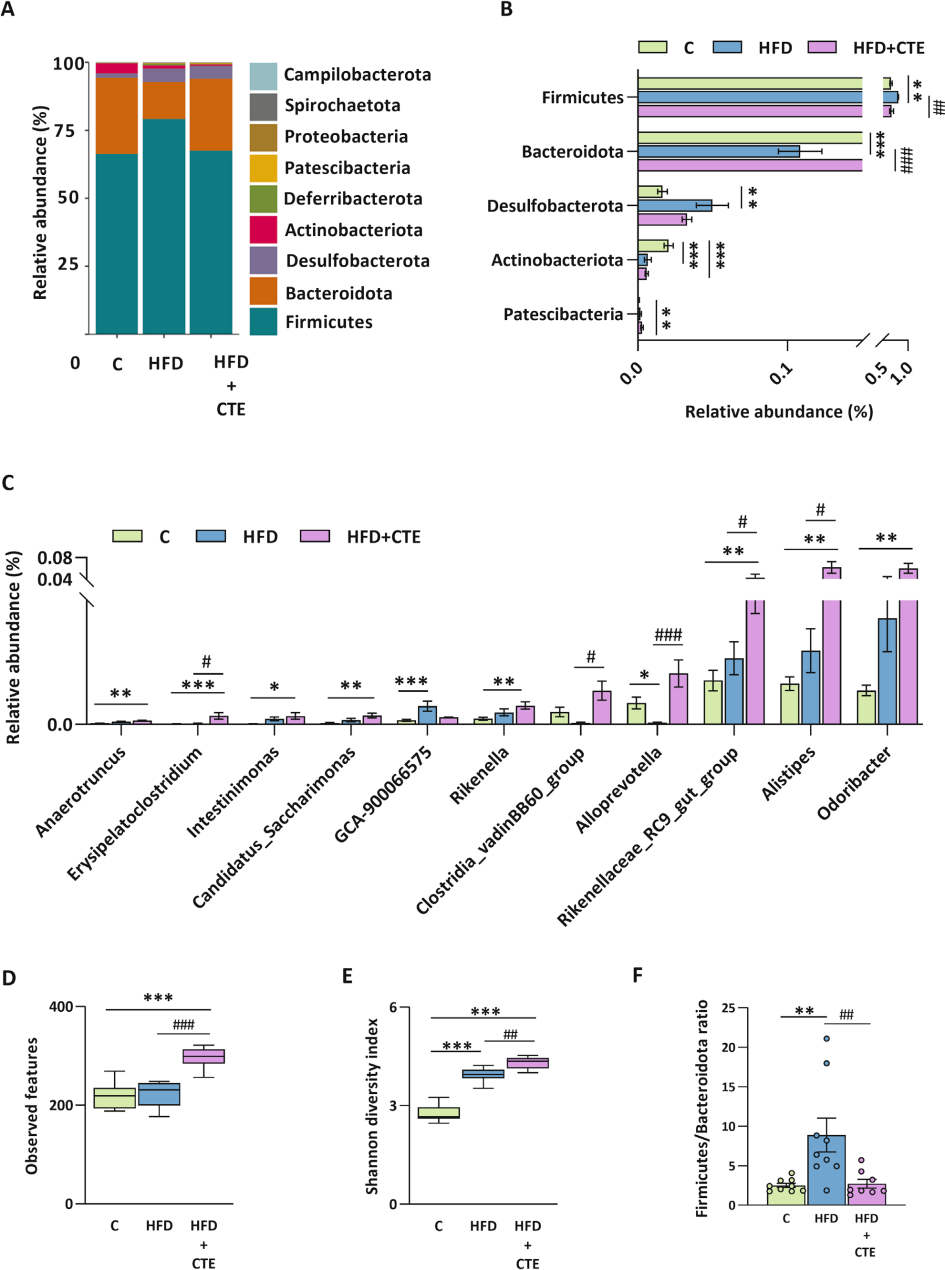
The effects of high‐fat diet and CTE on gut microbiota composition and alpha diversity. Gut microbiota composition and alpha diversity were analyzed in control group (C), high‐fat diet group (HFD) and high‐fat diet with *Crocus sativus* tepals extract group (HFD + CTE). (A) Bar chart of relative abundance of bacterial phyla; (B) Differential abundance of bacterial phyla; (C) Relative abundance of selected bacterial genera; (D, E) Alpha diversity indices: (D) Observed features and (E) Shannon diversity index; (F) *Firmicutes/Bacteroidota* ratio. One‐way ANOVA followed by a Tukey's *post hoc* test or a non‐parametric Kruskal–Wallis *H* test followed by a Dunn's *post hoc* test was used to assess statistical significance. The asterisk (*) indicates significant differences between the treatment groups compared to C group (*p < 0.05, **p < 0.01, ***p < 0.001), while the hash sign (#) indicates significant differences between HFD vs. HFD + CTE (#*p* < 0.05, ##*p* < 0.01, ###*p* < 0.001).

As shown in Figure 1A, the relative abundance of bacterial phyla differed significantly between experimental groups, although CTE treatment shifted the gut microbiota composition towards the one observed in the control animals. As expected, a high‐fat diet led to a marked decrease in *Bacteroidota* (*p* < 0.001) and an increase in *Firmicutes* (*p* < 0.01) phyla compared to the control group (Figure 1B). Supplementation with CTE reversed these alterations, significantly increasing *Bacteroidota* abundance (*p* < 0.001) and reducing *Firmicutes* (*p* < 0.01), compared to the HFD group (Figure 1B). Moreover, the relative abundance of *Patescibacteria* was significantly elevated in the CTE‐treated HFD group compared to the control mice (*p* < 0.01, Figure 1B), while high‐fat diet alone led to a significant increase in *Desulfobacterota* abundance compared to the control group (*p* < 0.01). The high‐fat diet also caused a reduction in *Actinobacteriota*, regardless of CTE treatment, compared to controls (*p* < 0.001, Figure 1B).

At the genus level (Figure 1C), supplementation with CTE led to a significant increase in the abundance of *Erysipelatoclostridium* (*p* < 0.001), *Intestinimonas* (*p* < 0.05), *Anaerotruncus*, *Candidatus Saccharimonas, Rikenella*, *Rikenellaceae RC9 gut group*, *Alistipes*, and *Odoribacter* (*p* < 0.01), compared to the control group. Moreover, compared to the HFD group (Figure 1C), CTE supplementation significantly increased the abundance of *Erysipelatoclostridium*, *Clostridia vadin BB60 group, Rikenellaceae RC9 gut group*, and *Alistipes* (*p* < 0.05), as well as *Alloprevotella* (*p* < 0.001). On the other hand, a high‐fat diet without CTE treatment caused an increase in *GCA900066575* (*p* < 0.001) and a decrease in *Alloprevotella* (*p* < 0.05) abundance compared to the controls (Figure 1C).

Alpha‐diversity indices further support the findings about modulatory effects of CTE on gut microbiota composition. The number of observed features (Figure 1D) indicated that CTE supplementation significantly enhanced microbial richness compared to both control and HFD group (*p* < 0.001). Similarly, the Shannon diversity index (Figure 1E) revealed increased richness and evenness in the HFD group relative to the control (*p* < 0.001), with an additional significant increase observed in the HFD + CTE group compared to the HFD (*p* < 0.01). Furthermore, the *Firmicutes/Bacteroidota* ratio (Figure 1F) was significantly elevated in the HFD group compared to the control (*p* < 0.01), which is a hallmark of diet‐induced dysbiosis, while CTE supplementation reduced this ratio compared to high‐fat diet alone (*p* < 0.01), suggesting a corrective shift towards a more balanced microbial composition.

**FIGURE 2:**
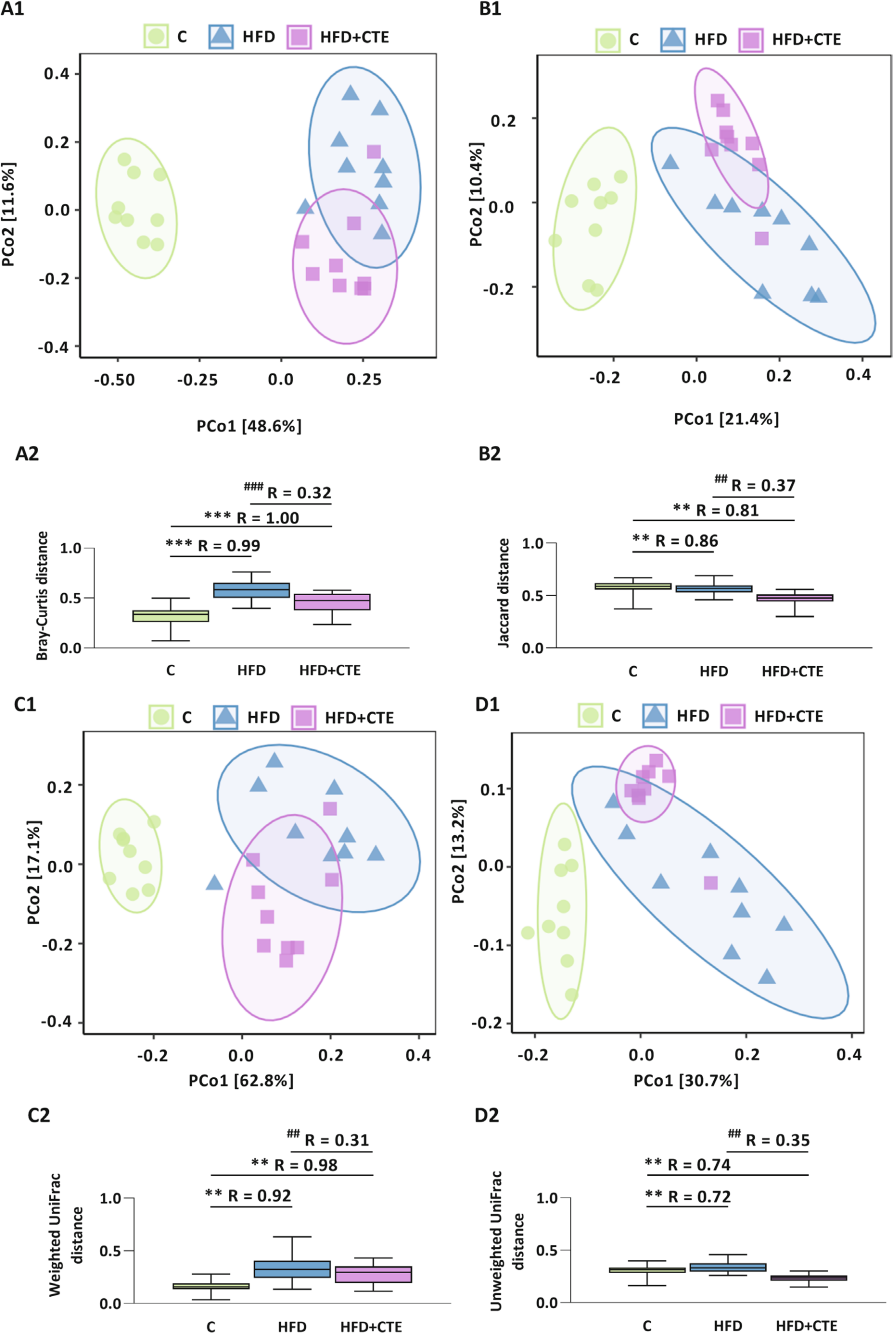
The effects of high‐fat diet and CTE on beta diversity of gut microbiota. Principal coordinates analysis (PCoA) plots based on (A1) Bray–Curtis, (B1) Jaccard, (C1) Weighted UniFrac and (D1) Unweighted UniFrac distance metrics and ANOSIM analysis of the same beta‐diversity metrics ((A2) Bray–Curtis, (B2) Jaccard, (C2) Weighted UniFrac, (D2) Unweighted UniFrac) in control group (C), high‐fat diet group (HFD) and high‐fat diet and *Crocus sativus* tepals extract group (HFD + CTE). The asterisk (*) indicates significant differences between the treatment groups compared to C group (**p < 0.01, ***p < 0.001), while the hash sign (#) indicates significant differences between HFD vs. HFD + CTE (##*p* < 0.01, ###*p* < 0.001).

Beta‐diversity analysis (Figure 2) using Bray‐Curtis, Jaccard, Weighted UniFrac and Unweighted UniFrac distances demonstrated distinct clustering of microbiota profiles between experimental groups. In all PCoA plots (Figure 2A1–D1), high‐fat diet induced a substantial shift in microbial community structure relative to the control, while supplementation with CTE additionally changed microbiota compared to the HFD group alone. These findings were supported by ANOSIM analysis (Figure 2A2–D2). Bray‐Curtis distances revealed increased R values in both HFD and HFD + CTE groups compared to the control (*p* < 0.001, Figure 2A2), indicating a divergence in microbial community structure, while the HFD + CTE group exhibited lower R values than the HFD group alone (*p* < 0.001, Figure 2A2). Similarly, Jaccard (Figure 2B2), Weighted UniFrac (Figure 2C2) and Unweighted UniFrac (Figure 2D2) distances confirmed that high‐fat diet significantly altered microbial composition in both groups regardless of treatment compared to the control (*p* < 0.01), whereas CTE supplementation additionally induced changes in R values compared to the HFD group without treatment (*p* < 0.01, Figure 2B2–D2).

### 3.3 CTE Improves Gut Morphology and Reduces Intestinal Permeability in Mice Fed High‐Fat Diet

**FIGURE 3:**
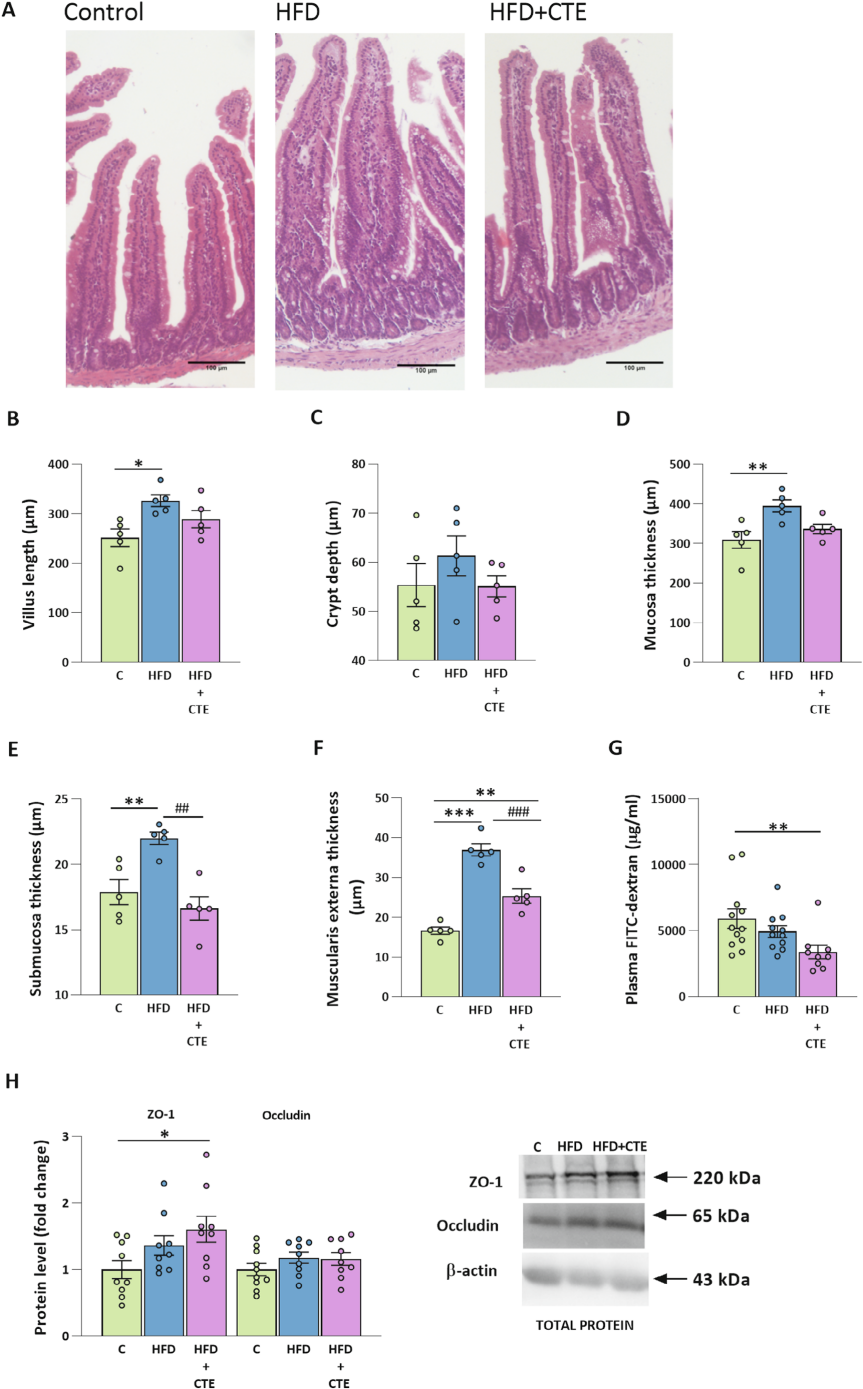
The effects of CTE on the morphology of jejunum, gut permeability and tight junction protein expression in mice fed high‐fat diet. (A) Representative micrographs of hematoxylin–eosin stain sections of the jejunum tissue in control group (C), high‐fat diet group (HFD) and high‐fat diet with *Crocus sativus* tepals extract group (HFD + CTE). The magnification was 10 × and scale bars are 100 μm. (B–F) Morphometric analysis of jejunum: (B) villus length, (C) crypt depth, (D) mucosa thickness, (E) submucosa thickness and (F) muscularis externa thickness. (G) FITC‐dextran permeability assay and (H) protein levels of ZO‐1 and occludin with representative Western blot images. All protein levels were measured in the total protein extract of the jejunum and normalized to β‐actin. Data are presented as mean ± SEM. One‐way ANOVA followed by a Tukey's *post hoc* test or a non‐parametric Kruskal–Wallis *H* test followed by a Dunn's *post hoc* test was used to assess statistical significance. The asterisk (*) indicates significant differences between the treatment groups compared to C group (*p < 0.05, **p < 0.01, ***p < 0.001), while the hash sign (#) indicates significant differences between HFD vs. HFD + CTE (##*p* < 0.01, ###*p* < 0.001).

Histological analysis of jejunum sections revealed pronounced morphological changes among the experimental groups (Figure 3A). In the HFD group, villi were longer (*p* < 0.05, Figure 3B) with increased mucosal and submucosal thickness compared to the controls (*p* < 0.01, Figure 3D,E, respectively). Additionally, the thickness of the muscularis externa was significantly increased in both HFD and HFD + CTE groups when compared to controls (HFD vs. C, *p* < 0.001; HFD + CTE vs. C, *p* < 0.01, Figure 3F). However, supplementation with CTE significantly reduced submucosal (*p* < 0.01, Figure 3E) and muscularis externa thickness (p < 0.001, Figure 3F) compared to the HFD group. As expected, the protective effect of CTE against increased intestinal permeability was also confirmed, as CTE administration significantly lowered plasma FITC‐dextran levels compared to the control animals (*p* < 0.01, Figure 3G). Furthermore, the protein level of the tight junction marker ZO‐1 was significantly elevated in the HFD + CTE group compared to the control group (*p* < 0.05), while occludin level remained unchanged (Figure 3H).

### 3.4 Oral Administration of CTE Has No Effect on Oxidative Status but Modulates Inflammation in the Jejunum

**FIGURE 4:**
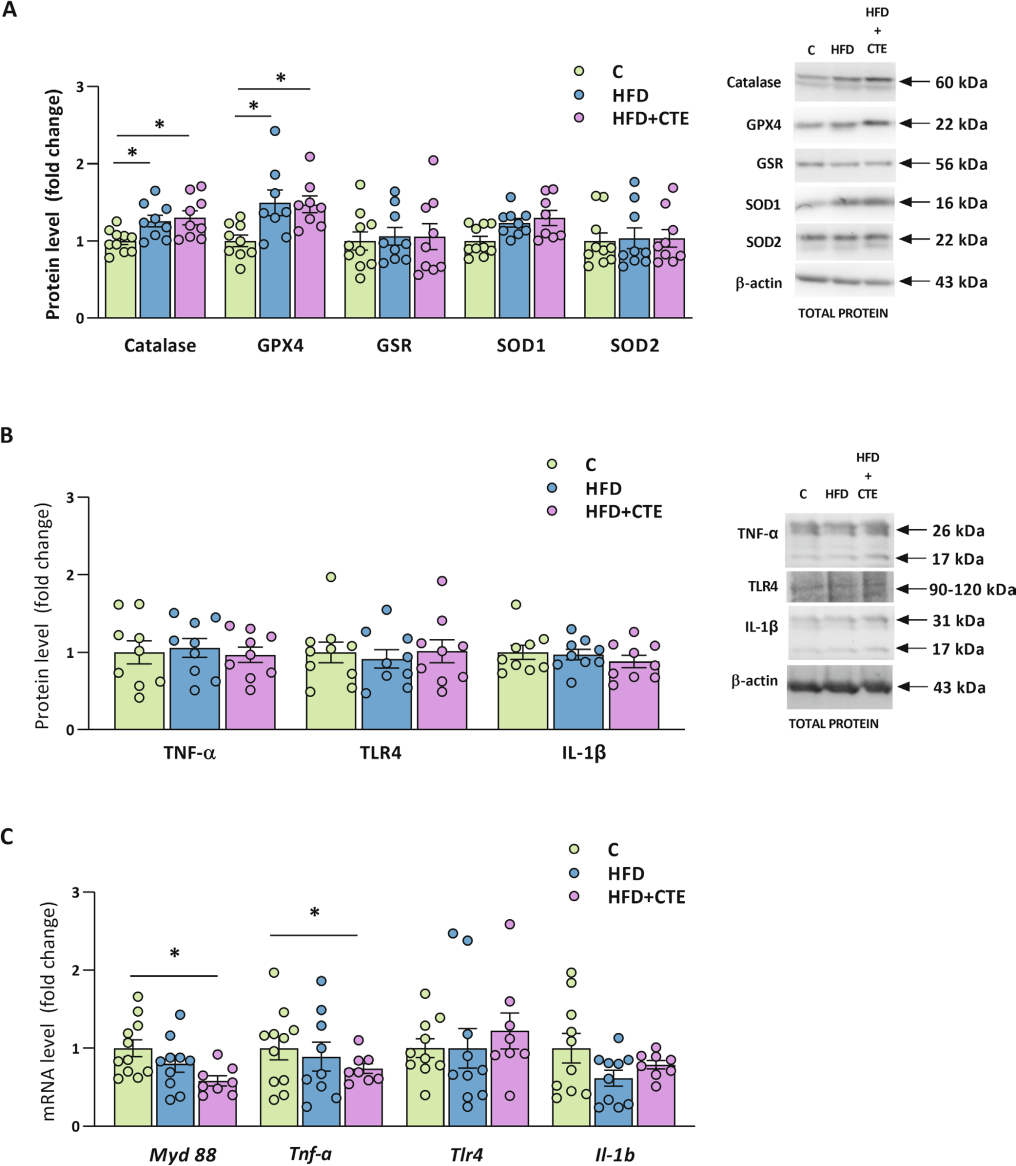
The effects of high‐fat diet and CTE on intestinal oxidative stress and inflammatory markers. (A) Protein levels of antioxidant enzymes: Catalase, GPX4, GSR, SOD1 and SOD2 with representative Western blot images shown on the right; (B) Protein levels of TNF‐α, TLR4 and IL‐1β with representative Western blot images shown on the right. Both the precursor and soluble forms of TNF‐α (26 kDa and 17 kDa, respectively) and IL‐1β (31 kDa and 17 kDa, respectively) were detected. To quantify total TNF‐α and IL‐1β, the densitometry values of the bands for both forms were summed and shown in the graph; (C) mRNA levels of inflammatory markers *Myd88*, *Tnf‐* *α*, *Tlr4*, and *Il‐1b* in the jejunum tissue of control group (C), high‐fat diet group (HFD) and high‐fat diet with *Crocus sativus* tepals extract group (HFD + CTE). All protein levels were measured in the total protein extract of the jejunum and normalized to β‐actin. The data are presented as mean ± SEM. The gene expression assessed by qPCR was normalized to *Hprt*. One‐way ANOVA followed by a Tukey's *post hoc* test or a non‐parametric Kruskal–Wallis *H* test followed by a Dunn's *post hoc* test was used to assess statistical significance. The asterisk (*) indicates significant differences between the treatment groups compared to C (*p < 0.05).

To assess the oxidative status in the intestine, we measured the protein levels of key antioxidant enzymes in the jejunum tissue. Catalase and GPX4 levels were significantly increased in both HFD groups, regardless of the CTE supplementation (*p* < 0.05, Figure 4A). However, no significant differences between the experimental groups were detected in the protein levels of GSR, SOD1, and SOD2 enzymes (Figure 4A).

In parallel, we examined protein and mRNA levels of TNF‐α, TLR4 and IL‐1β in the jejunum. Both the precursor and soluble forms of TNF‐α (26 kDa and 17 kDa, respectively) [49] and IL‐1β (31 kDa and 17 kDa, respectively) [50] were detected. To obtain total TNF‐α and IL‐1β values, the densitometry values of the bands for both forms were summed. As shown in Figure 4B, total protein levels of TNF‐α, TLR4, and IL‐1β were unchanged in all experimental groups. However, CTE administration led to significant reduction of mRNA levels of *Myd88* and *Tnf‐* *α* in the HFD + CTE group compared to the control (*p* < 0.05). The expression of *Tlr4* and *Il‐1b* was not changed between the experimental groups (Figure 4C).

### 3.5 Impact of CTE Administration on Hepatic Inflammation and Lipid Metabolism

**FIGURE 5:**
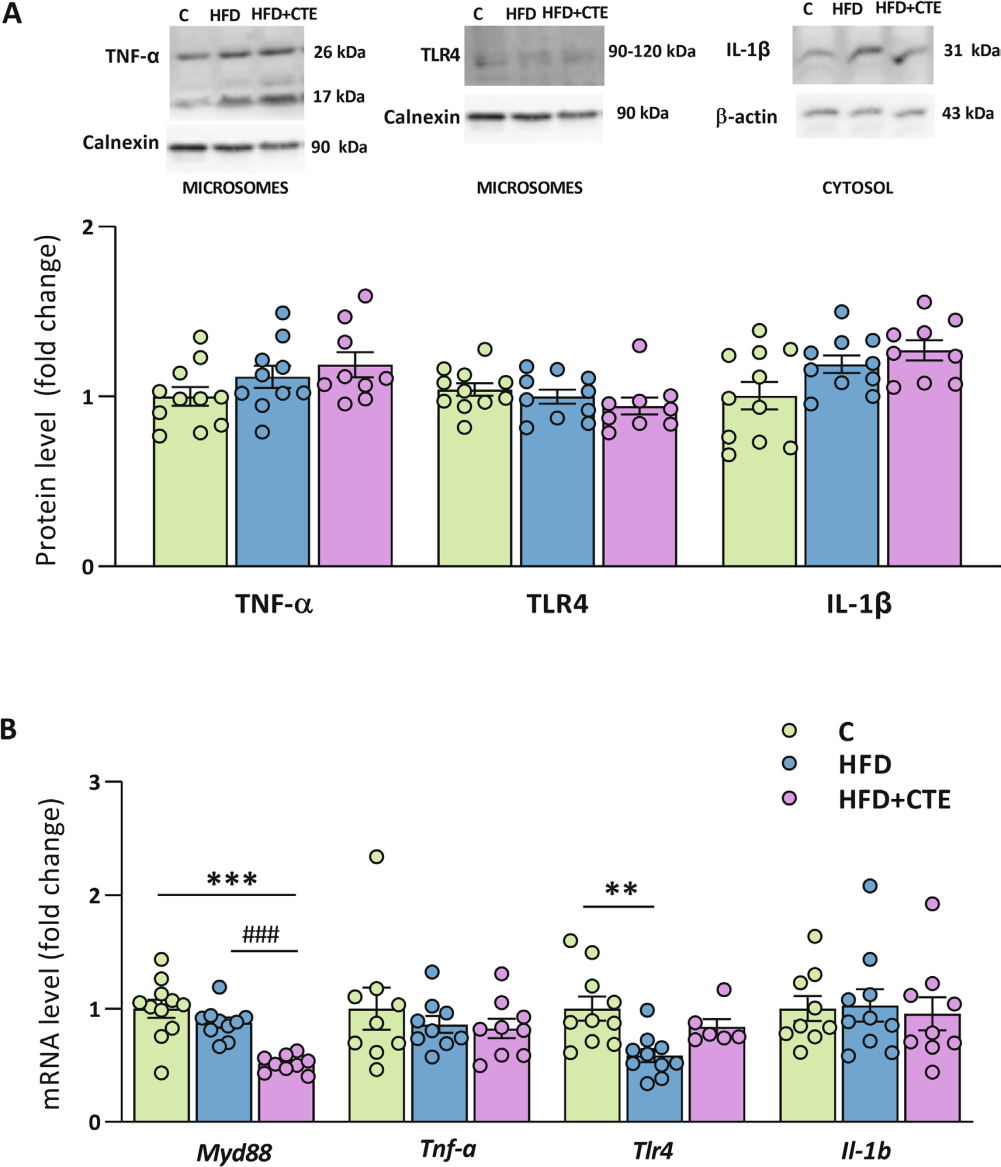
The effects of CTE on hepatic inflammation in mice fed high‐fat diet. (A) Protein levels of TNF‐α, TLR4 and IL‐1β, with representative Western blot images shown above graphs. TLR4 and TNF‐α (26 kDa membrane‐bound precursor and 17 kDa soluble form) were detected in the microsomal fraction, while IL‐1β was detected in the cytosol (31 kDa precursor form). For TNF‐α, the densitometry values of the 26 kDa and 17 kDa bands were summed to obtain the total TNF‐α protein level shown in the graph; (B) Relative mRNA levels of *Myd88*, *Tnf‐α*, *Tlr4*, and *Il‐1b* in the liver of control group (C), high‐fat diet group (HFD) and high‐fat diet with *Crocus sativus* tepals extract group (HFD + CTE). The gene expression assessed by qPCR was normalized to *Hprt* and protein levels were normalized to Calnexin in the microsomes and β‐actin in the cytosol. All data are presented as mean ± SEM. One‐way ANOVA followed by a Tukey's *post hoc* test or a non‐parametric Kruskal–Wallis *H* test followed by a Dunn's *post hoc* test was used to assess statistical significance. The asterisk (*) indicates significant differences between the treatment groups compared to C (**p < 0.01, ***p < 0.001), while the hash sign (#) indicates significant differences between HFD vs. HFD + CTE (###*p* < 0.001).

To investigate the effects of CTE on inflammatory markers in the liver, we measured the protein levels and gene expression of key pro‐inflammatory mediators: TNF‐α, TLR4, and IL‐1β. TLR4 and TNF‐α (26 kDa membrane‐bound precursor and 17 kDa soluble form) were detected in the microsomal fraction, while IL‐1β was detected in the cytosol (31 kDa precursor form). For TNF‐α, the densitometry values of the 26 kDa and 17 kDa bands were summed to obtain the total TNF‐α protein level. The results showed that the protein levels of TNF‐α, TLR4, and IL‐1β were not affected by any of the treatments (Figure 5A). However, CTE supplementation significantly reduced the mRNA level of *Myd88* compared to both the control and HFD groups (*p* < 0.001, Figure 5B). The mRNA levels of *Tnf‐* *α* and *Il‐1b* were not significantly affected by the diet or CTE treatment, while the mRNA level of *Tlr4* was significantly decreased in the HFD group relative to the control (*p* < 0.01, Figure 5B).

**FIGURE 6:**
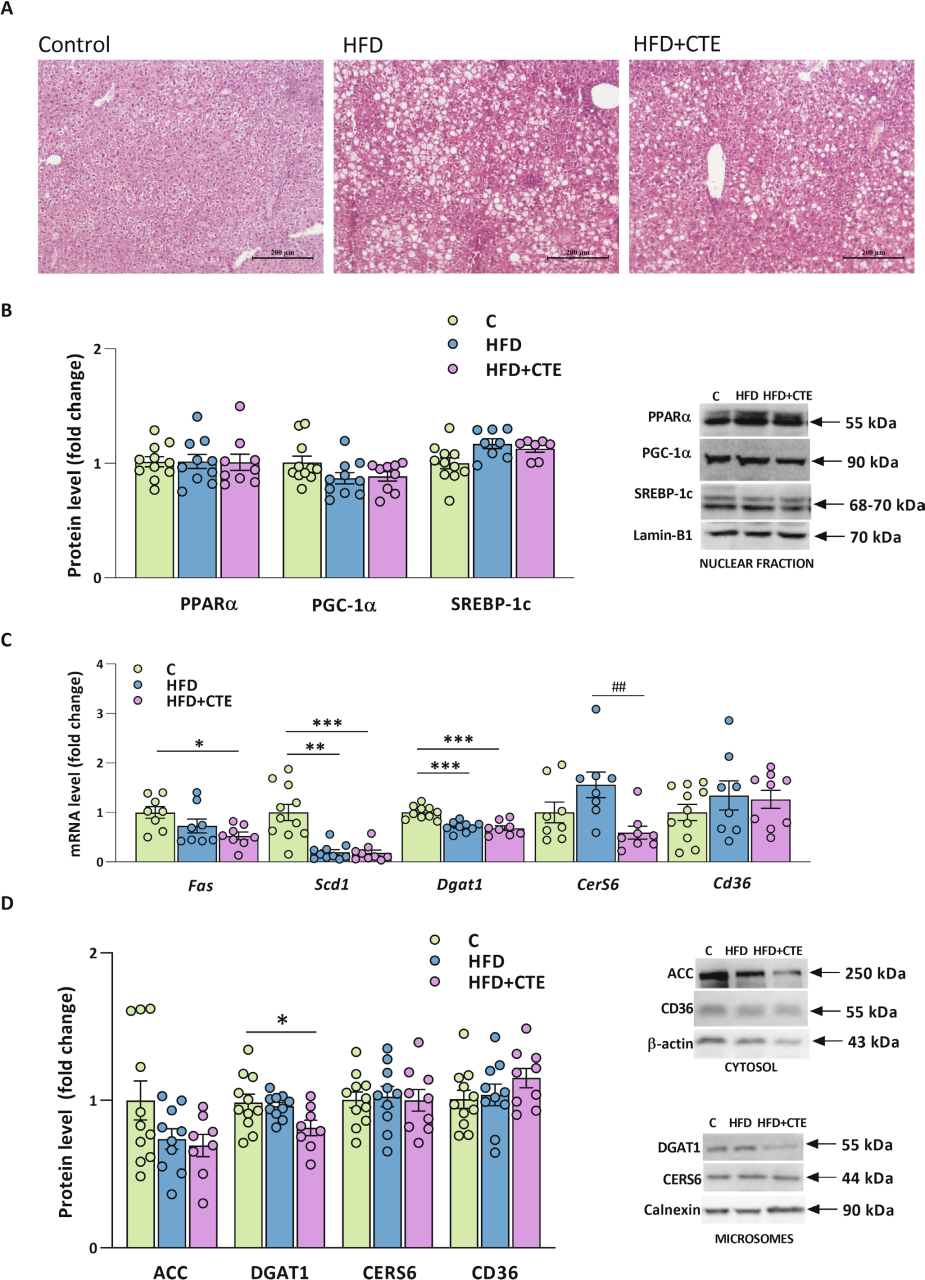
The effects of CTE on hepatic steatosis and lipid metabolism in mice fed high‐fat diet. (A) Representative micrographs of hematoxylin–eosin stained liver sections from the control group (C), high‐fat diet group (HFD) and high‐fat diet with *Crocus sativus* tepals extract group (HFD + CTE). The magnification was 10× and scale bars are 200 μm; (B) Protein levels of lipid metabolism regulators PPARα, PGC‐1α, and SREBP‐1c with representative Western blot images shown on the right; (C) Relative mRNA levels of genes involved in lipid metabolism: *Fas*, *Scd1*, *Dgat*1, *CerS6*, and *Cd36*. The gene expression assessed by qPCR was normalized to *Hprt*. (D) Protein levels of ACC and CD36 in the cytosol, and DGAT and CERS6 in the microsomal fraction, with representative Western blot images shown on the right. All examined protein levels were normalized to lamin‐B1 for the nuclear fraction, Calnexin for the microsomes, and β‐actin for the cytosol. All data are presented as mean ± SEM. One‐way ANOVA followed by a Tukey's *post hoc* test or a non‐parametric Kruskal–Wallis *H* test followed by a Dunn's *post hoc* test was used to assess statistical significance. The asterisk (*) indicates significant differences between the treatment groups compared to C (*p < 0.05, **p < 0.01, ***p < 0.001), while the hash sign (#) indicates significant differences between HFD vs. HFD + CTE (##*p* < 0.01).

Liver sections stained with hematoxylin and eosin showed that treatment with a high‐fat diet led to the development of steatosis without concomitant fibrosis, regardless of the CTE treatment (Figure 6A). In addition, we assessed the expression of key genes and proteins involved in lipid synthesis and uptake in the liver. No significant differences were observed in PPARα, PGC‐1α, and SREBP‐1c protein levels (Figure 6B) in the nuclear fraction. However, CTE supplementation significantly decreased *Fas* mRNA level (*p* < 0.05, Figure 6C). Additionally, mRNA levels of *Scd1* and *Dgat1* were reduced in both HFD and HFD + CTE groups compared to the control (*Scd1*: HFD vs. C, *p* < 0.01; HFD + CTE vs. C, *p* < 0.001; *Dgat1*: HFD vs. C and HFD + CTE vs. C, both *p* < 0.001, Figure 6C). Finally, CTE supplementation reversed the high‐fat diet‐induced increase in mRNA level of *CerS6* (*p* < 0.01), while mRNA level of *Cd36* remained unchanged in all experimental groups (Figure 6C). As shown in Figure 6D, DGAT1 protein level in the microsomal fraction was significantly decreased with CTE treatment (*p* < 0.05), while other examined proteins involved in hepatic lipid metabolism, ACC and CD36 in the cytosol, and the CERS6 in the microsomal fraction, remained unchanged in all experimental groups.

## 4 Discussion

Our study is among the first to demonstrate that orally administered CTE affects hepatic lipid metabolism and inflammation and restores high‐fat diet‐induced gut dysbiosis. These findings are evidenced by suppressed expression of *Fas* and *CerS6* and decreased *MyD88* inflammatory marker in the liver and the shift in the gut microbiota composition towards the one observed in control animals. CTE treatment also improved gut barrier integrity, as confirmed by reduced intestinal permeability and downregulation of pro‐inflammatory markers in the jejunum. The improvement in the microbiota‐gut‐liver axis was accompanied by ameliorations in blood parameters, including lower serum FFA levels and decreased circulating BHB.

We have previously shown that our animal model of high‐fat diet‐induced obesity exhibits increased caloric intake, total body mass and white adipose tissue accumulation [41], while these mice also develop hepatic steatosis, the first stage of MASLD. Given the anatomical and functional connection between the liver and the gut [51], we also analyzed the composition of the gut microbiota in this model. Consistent with previous findings, we confirmed that high‐fat diet profoundly altered the composition of the gut microbiota, characterized by a decrease in *Bacteroidota* and an increase in *Firmicutes* phyla, a commonly observed feature of obesity‐associated dysbiosis [52, 53]. Importantly, supplementation with CTEs effectively reversed these changes and returned the *Firmicutes/Bacteroidota* ratio to control levels, suggesting a possible modulatory role of the extracts in mitigating gut dysbiosis induced by high‐fat diet. However, the persistent reduction in *Actinobacteriota* across HFD groups, regardless of CTE supplementation, suggests that some aspects of high‐fat diet‐induced dysbiosis may be less amenable to dietary intervention [54]. At a more detailed taxonomic level, CTE administration increased the relative abundance of several genera known for producing short‐chain fatty acids (SCFAs) and exerting anti‐inflammatory effects, including *Intestinimonas*, *Anaerotruncus*, *Alistipes* and *Odoribacter* [55, 56]. In addition, treatment with the extract resulted in a significant increase in the relative abundance of genera *Alistipes*, *Rikenellaceae RC9 gut group*, *Alloprevotella* and *Clostridia vadin BB60 group* compared to the HFD group. The increase of genus *Alistipes and Rikenellaceae RC9 gut group*, both of which are reduced in obese individuals and have been implicated in gut barrier integrity and the production of anti‐inflammatory mediators [57, 58], further reinforces the potential of CTE in preserving gut health under obesogenic conditions. The genus *Alloprevotella* is known for its ability to ferment complex polysaccharides and produce SCFAs such as acetate and propionate, which are key metabolites with beneficial effects on host energy metabolism and immune modulation [56]. Similarly, the *Clostridia vadin BB60 group*, although less well‐characterized, has been identified as a SCFA‐producing group inversely correlated with obesity, dyslipidemia and insulin resistance in mice fed high‐fat diet [59]. Thus, the observed enrichment of *Alloprevotella* and *Clostridia vadin BB60 group* following the CTE treatment may reflect a shift towards a microbial composition associated with improved metabolic outcomes.

Alpha‐diversity metrics further corroborate potential microbiota‐restorative effects of CTE as microbial richness and evenness were significantly enhanced in the CTE‐supplemented animals, surpassing even the control levels for some indices. This finding aligns with previous reports showing that a higher alpha diversity is widely recognized as a hallmark of a healthy microbial ecosystem [60]. In addition, beta‐diversity analyses revealed that high‐fat diet induced substantial shifts in microbial composition, as demonstrated by distinct clustering patterns in all PCoA plots. However, CTE supplementation further altered the microbial profiles, as corroborated by ANOSIM‐based comparisons, which showed significantly lower R values in the HFD + CTE relative to the HFD group. Such findings indicate reduced intergroup dissimilarity and a partial restoration of the gut microbiota composition.

Our findings are consistent with previous reports demonstrating the ability of dietary polyphenols and flavonoids to beneficially modulate gut microbiota composition and counteract diet‐induced dysbiosis under obesogenic conditions [61]. U‐HPLC–HRMS analysis of the hydroalcoholic CTE used in this study identified 131 phenolic compounds, including flavonoids (anthocyanins, dihydrochalcones, flavanols, isoflavonoids), phenolic acids (hydroxybenzoic, hydroxycinnamic, and hydroxyphenylpropanoic acids), stilbenes, and other polyphenols [41]. Anthocyanins are the main class of bioactive compounds in the CTE and are responsible for the characteristic coloration of tepals. Previous studies have shown that anthocyanins such as peonidin 3‐O‐arabinoside, cyanidin‐3‐glucoside, delphinidin‐3‐rutinoside, and malvidin‐3‐glucoside, which are abundant in CTE, play a key role in modulating gut microbiota composition and intestinal inflammation [62, 63, 64, 65]. Due to their limited absorption in the upper gastrointestinal tract, a substantial proportion of anthocyanins reaches the cecum and colon, where they undergo microbial biotransformation into smaller phenolic metabolites (e.g., syringic, p‐coumaric, vanillic, and 4‐hydroxybenzoic acids). These reactions are mediated by bacterial enzymes such as β‐glucosidase, mainly expressed by *Bifidobacterium* spp. and *Lactobacillus* spp., thereby promoting the growth of beneficial taxa (*Bifidobacterium, Lactobacillus, Enterococcus, Eggerthella lenta*) and counteracting gut dysbiosis [64]. The anti‐inflammatory activity of anthocyanins, particularly cyanidin‐3‐glucoside and delphinidin‐3‐rutinoside, is closely linked to these microbiota‐dependent transformations, resulting in reduced NF‐κB p65 activation and decreased expression of pro‐inflammatory mediators (IL‐6, IL‐8, IL‐1β, TNF‐α, COX‐2) [63]. The second most abundant compound in the extract is epigallocatechin 7‐O‐glucuronide, along with other flavanols, such as epicatechin 3‐O‐gallate. There is growing evidence that these compounds also modulate gut microbiota by increasing beneficial bacteria (e.g., *Akkermansia*, *Bifidobacterium*) and reducing harmful ones such as *Desulfovibrio*. These flavanols and their metabolites contribute to enhanced gut barrier integrity, partly through SCFA production and activation of the aryl hydrocarbon receptor (AhR) pathway, and exert anti‐inflammatory effects by suppressing TLR4/NF‐κB signaling. Therefore, the effects of CTE observed in our study are likely due to the combined contribution of several bioactive polyphenolic compounds, whose presence supports the modulation of gut microbiota, intestinal barrier function, and inflammatory pathways.

Furthermore, it has been shown that factors such as diet and microbiota composition can influence villus length and mucosal properties of the small intestine, including the jejunum [66, 67, 68, 69]. In the present study, high‐fat diet led to pronounced morphological changes in the jejunum, such as an increase in villus length and mucosal thickness as well as an expansion of the submucosa and muscularis externa. Some studies have reported that intestinal dysbiosis primarily affects the integrity of the mucosa, resulting in villous atrophy or thinning of the mucosa [70], while others, including ours, showed that the observed structural changes may reflect compensatory adaptations to the increased nutrient uptake by the high‐fat diet [71, 72]. It is noteworthy that the present study also showed that CTE supplementation reversed these intestinal changes induced by high‐fat diet, as it significantly reduced the thickness of the submucosa and muscularis externa. Furthermore, CTE significantly reduced intestinal permeability in mice fed high‐fat diet, as evidenced by reduced translocation of FITC‐dextran into the bloodstream. These effects on intestinal permeability can possibly be attributed to CTE‐induced changes in the composition of the gut microbiota, particularly the increase in SCFA‐producing bacteria such as *Intestinimonas*, *Anaerotruncus*, *Alistipes*, and *Odoribacter*. In addition, the improved intestinal barrier function could be a consequence of increased regeneration of the epithelium and higher concentrations of tight junction proteins such as ZO‐1 and occludin [73]. Indeed, in the present study, we observed that supplementation with CTE significantly increased ZO‐1 protein levels.

Extracts from *Crocus sativus* tepals are already known for their antioxidant properties [38, 74], and in line with this we investigated whether these extracts could have a similar effect in the jejunum. However, we only observed increased protein levels of catalase and GPX4 in the jejunum of both HFD groups, independent of CTE supplementation, which might indicate an adaptive response to increased oxidative stress associated with high energetic substrate availability [75]. Nonetheless, we confirmed that supplementation with CTE significantly reduced mRNA levels of intestinal *Myd88* and *Tnf‐α*, highlighting its anti‐inflammatory potential. Although TLR4 remained unchanged in the intestine, the observed downregulation of *Myd88* mRNA indicates that the applied treatment selectively suppressed downstream TLR4/MyD88 signaling, suggesting an early anti‐inflammatory effect even without changes at the TLR4 receptor level. This aligns with the observed decrease of *Tnf‐α* mRNA level, which can also be considered anti‐inflammatory, despite unchanged TNF‐α protein level. Namely, cytokine mRNAs are subject to extensive post‐transcriptional and translational regulation [76, 77, 78], which can buffer protein production and delay changes in protein abundance relative to transcriptional changes. Therefore, a decrease in cytokine transcripts is consistent with an anti‐inflammatory effect, even in the absence of immediate changes in protein levels. This is particularly important in the context of the gut microbiota, which plays a central role in regulation of immune response in the gut [79], partly through the activity of the SCFAs produced by specific bacterial genera. As previously mentioned, CTE supplementation increased the relative abundance of several bacterial genera associated with anti‐inflammatory effects through increased SCFA production [56, 80, 81, 82].

Since the intestine and liver are anatomically and functionally connected via the portal circulation and form the gut‐liver axis, disturbances in gut homeostasis can facilitate the translocation of microbial products and thus influence the immune and metabolic responses of the liver [83, 84]. Therefore, we analyzed the hepatic inflammatory response and lipid metabolism. The results showed unchanged protein levels of TNF‐α, TLR4, and IL‐1β, while changes at the mRNA levels were observed. Specifically, a high‐fat diet led to a significant downregulation of the pro‐inflammatory marker *Tlr4* in the liver, while *Myd88* was downregulated in the HFD groups regardless of CTE supplementation. In contrast to the gut, in the liver, chronic metabolic stress induced by high‐fat diet together with increased endotoxins from the gut can lead to sustained activation of the Myd88 signaling pathway. As a compensatory response to metabolic stress, liver cells downregulate *Myd88* expression to protect themselves from excessive inflammation and tissue damage [85, 86].

As mentioned above, the high‐fat diet applied in this study led to the development of hepatic steatosis, while liver mass, liver‐to‐body mass ratio and liver enzymes ALT, AST and GGT remained unchanged. As expected, total cholesterol levels were significantly elevated by high‐fat diet [87], but this effect was independent of the CTE supplementation. Indeed, most lipid‐lowering effects reported for the *Crocus sativus* plant are attributed to the stigmas, primarily because they contain bioactive compounds such as crocin, crocetin, safranal, and picrocrocin [88]. In contrast, as previously mentioned, tepals are mainly rich in flavonoids (kaempferol and anthocyanins) and phenolic acids, which are compounds with proven antioxidant and anti‐inflammatory properties [38, 89, 90]. This suggests that the phytochemical profile of CTE primarily targets oxidative and inflammatory modulation rather than lipid homeostasis. In line with this, at the histological level, hepatic steatosis (although reversible) was not affected by oral supplementation with CTE after 5‐week treatment, which was also consistent with the unchanged protein levels of PPARα, PGC‐1α, and SREBP‐1c. However, mRNA levels of their downstream targets, *Scd1* and *Dgat1*, were significantly decreased after a high‐fat diet, while DGAT protein levels decreased with CTE treatment. These findings suggest that hepatocytes may employ feedback mechanisms to limit further lipid accumulation and lipotoxicity under sustained lipid excess induced by high‐fat diet [91]. Interestingly, the downregulation of *Fas* was observed exclusively in the CTE‐supplemented group, as well as significant downregulation of *CerS6* at the mRNA level, an enzyme responsible for the synthesis of C16 ceramides, which play a role particularly in insulin resistance and hepatocellular stress [92]. These changes were not accompanied by decreased protein levels of ACC. The reduction of *Fas* mRNA level, despite unchanged ACC expression, suggests a selective downregulation of the terminal steps of *de novo* lipogenesis. ACC activity is often controlled post‐translationally, whereas FAS is more transcriptionally sensitive and its decreased expression may therefore reflect reduced fatty acid synthesis capacity and improved metabolic and/or inflammatory status. Therefore, the observed reduction in *CerS6* expression together with the downregulation of *Fas* suggests a potential protective mechanism by which CTE may alleviate high‐fat diet‐induced metabolic disturbances in the liver. In addition, CTE decreased serum FFA and BHB levels, which may be a marker of normalized liver metabolism [93]. Possible mechanisms leading to lower FFA and BHB levels could be improved insulin action after treatment with CTE, which was shown in our previous study [41], but also modulation of the gut microbiota, which can lead to better energy homeostasis. Indeed, the gut microbiota analysis data showed an increased abundance of genera that produce SCFA, of which butyrate and propionate in particular are known to act as signaling molecules that inhibit adipose tissue lipolysis [94, 95]. This is also consistent with our previous data showing that CTE reduces lipolytic capacity in subcutaneous adipose tissue in the same animals [41].

Our results provide convincing evidence that CTE, when administered *per os*, has beneficial effects on metabolic disturbances induced by a high‐fat diet. First of all, CTE treatment restored the composition of the gut microbiota, particularly promoting SCFA‐producing and anti‐inflammatory bacterial genera. It also improved the integrity of the gut barrier and reduced inflammation in the jejunum. These improvements in the gut and microbiota were accompanied by a suppression of *Fas* and *CerS6* expression in the liver and a reduction in circulating FFA and BHB levels. Overall, these changes highlight a possible link between the gut microbiota and the metabolic benefits of treatment with CTE, suggesting its therapeutic potential for the prevention or treatment of metabolic disorders associated with obesity.

## Author Contributions

**Biljana Bursać:** investigation, formal analyses, writing. **Miloš Vratarić:** investigation, formal analyses, writing. **Ljupka Gligorovska:** visualization, formal analyses. **Luisa Bellachioma:** investigation, formal analyses. **Ana Teofilović:** conceptualization, writing – review and editing. **Danijela Vojnović Milutinović:** conceptualization, writing – review and editing. **Camilla Morresi:** investigation, formal analyses. **Elisabetta Damiani:** conceptualization, writing – review and editing. **Tiziana Bacchetti:** conceptualization, writing – review and editing. **Ana Djordjevic:** conceptualization, supervision, writing – review and editing. All authors: final approval of the submitted version.

## Funding

This work was supported by the Ministry of Science, Technological Development and Innovation of the Republic of Serbia under Grant No. 451‐03‐136/2025‐03/200007 and by ordinary funds of the Polytechnic University of Marche granted to T.B. and E.D. The funders had no role in the design, analysis, or writing of this article. The results presented in this manuscript are in line with Sustainable Development Goal 3 (Good Health and Well‐being) of the United Nations 2030 Agenda.

## Conflicts of Interest

The authors declare no conflicts of interest.

## Data Availability

The data that support the findings of this study are available from the corresponding author upon reasonable request.
